# Supplementary material for: Effects of Compression on the Local Iodine Environment in Dipotassium Zinc Tetraiodate(V) Dihydrate K2Zn(IO3)4·2H2O
Source: Inorg Chem. 2025 Apr 10;64(15):7784–96. doi: 10.1021/acs.inorgchem.5c00911 (PMC12124715; doi:10.1021/acs.inorgchem.5c00911)
Supplement: Supplementary file 1 [file ic5c00911_si_001.pdf]

Supporting Information

Effects of Compression on the Local Iodine Environment in Dipotassium Zinc Tetraiodate(V) Dihydrate $K_2Zn(IO_3)_4·2H_2O$

Daniel Errandonea[1,*], Robin Turnbull[1], Hussien H.H. Osman[1,2], Zoulikha Hebboul[3], Pablo Botella[1], Neha Bura[1], Peijie Zhang[1], Jose Luis Rodrigo Ramon[1], Josu Sanchez-Martin[1], Catalin Popescu[4], Francisco J. Manjon[2]

[1]Departamento de Física Aplicada-ICMUV-MALTA Consolider Team, Universitat de Valencia, 46100 Valencia, Spain

[2]Instituto de Diseño para la Fabricación y Producción Automatizada, MALTA Consolider Team, Universitat Politècnica de València, 46022 València, Spain

[3]Laboratoire Physico-Chimie des Matériaux, Université Amar Telidji de Laghouat, BP 37G, Route de Ghardaia, Laghouat 03000, Algeria

[4]CELLS-ALBA Synchrotron Light Facility, Cerdanyola 08290, Barcelona, Spain; https://orcid.org/0000-0001-6613-4739; Email: cpopescu@cells.es

*Corresponding author, Email: daniel.errandonea@uv.es

Table S1: Lattice parameters determined from experiments at different pressures in run 1.

Pressure (GPa)	*a* (Å)	*b* (Å)	*c* (Å)	β (°)
10^{-4}	8.286(6)	7.728(5)	11.086(8)	90.30(1)
0.40(5)	8.255(6)	7.669(5)	11.053(8)	90.38(1)
0.90(5)	8.216(6)	7.613(5)	11.030(8)	90.45(1)
1.20(5)	8.196(6)	7.579(5)	11.014(8)	90.49(1)
1.25(5)	8.199(6)	7.570(5)	11.007(8)	90.49(1)
2.00(5)	8.136(6)	7.475(5)	10.943(8)	90.62(1)
2.35(5)	8.118(6)	7.445(5)	10.918(8)	90.67(1)
2.90(5)	8.081(6)	7.390(5)	10.877(8)	90.75(1)
3.70(5)	8.045(6)	7.337(5)	10.835(8)	90.83(1)
4.20(5)	8.036(6)	7.311(5)	10.814(8)	90.85(1)
4.50(5)	8.014(6)	7.286(5)	10.794(8)	90.93(1)
5.00(5)	7.982(6)	7.250(5)	10.764(8)	91.00(1)
5.60(5)	7.968(6)	7.229(5)	10.742(8)	91.06(2)
6.10(5)	7.958(6)	7.215(5)	10.727(8)	91.09(2)
6.50(5)	7.945(6)	7.196(5)	10.710(8)	91.13(2)
6.70(5)	7.933(6)	7.183(5)	10.696(8)	91.14(2)
7.00(5)	7.926(6)	7.167(5)	10.685(8)	91.18(2)
7.40(5)	7.907(6)	7.147(5)	10.670(8)	91.22(2)
7.80(5)	7.892(6)	7.125(5)	10.650(8)	91.25(2)
8.80(5)	7.869(6)	7.090(5)	10.622(8)	91.32(2)
10.10(5)	7.843(6)	7.066(5)	10.594(8)	91.34(2)
10.55(5)	7.838(6)	7.057(5)	10.564(8)	91.36(2)

Pressure (GPa)	*a* (Å)	*b* (Å)	c (Å)	β (º)
1.60(5)	8.159(6)	7.536(5)	10.968(8)	90.69(1)
1.90(5)	8.137(6)	7.483(5)	10.944(8)	90.75(1)
2.90(5)	8.077(6)	7.415(5)	10.884(8)	90.79(1)
4.10(5)	8.031(6)	7.331(5)	10.825(8)	90.93(1)
5.50(5)	7.979(6)	7.240(5)	10.758(8)	91.06(1)
7.40(5)	7.911(6)	7.148(5)	10.682(8)	91.20(1)
8.95(5)	7.885(6)	7.085(5)	10.599(8)	91.31(1)
9.75(5)	7.852(6)	7.057(5)	10.57388)	91.34(1)
10.50(5)	7.851(6)	7.038(6)	10.576(8)	91.36(1)
10.85(5)	7.821(6)	7.029(6)	10.576(8)	91.36(2)
11.20(5)	7.818(6)	7.020(6)	10.578(8)	91.37(2)
11.40(5)	7.814(6)	7.014(6)	10.576(8)	91.39(2)
12.20(5)	7.791(6)	6.998(6)	10.539(8)	91.42(2)
12.80(5)	7.778(6)	6.985(6)	10.508(8)	91.42(2)
13.40(5)	7.772(6)	6.973(6)	10.502(8)	91.43(2)
13.90(5)	7.764(6)	6.960(6)	10.484(8)	91.43(2)
14.50(5)	7.758(6)	6.946(6)	10.472(8)	91.46(2)
15.10(5)	7.731(6)	6.933(5)	10.460(8)	91.50(2)
15.60(5)	7.721(6)	6.920(5)	10.435(8)	91.50(2)
15.90(5)	7.712(6)	6.912(5)	10.425(8)	91.50(2)
16.40(5)	7.704(6)	6.905(5)	10.408(8)	91.51(2)
16.85(5)	7.696(6)	6.898(6)	10.403(9)	91.51(2)
17.35(5)	7.689(6)	6.890(5)	10.398(9)	91.52(2)
17.90(5)	7.682(6)	6.881(5)	10.393(9)	91.52(2)
18.05(5)	7.678(6)	6.873(5)	10.388(9)	91.53(2)
18.45(5)	7.664(6)	6.861(5)	10.379(9)	91.54(2)
19.00(5)	7.658(6)	6.851(5)	10.369(9)	91.54(2)
19.55(5)	7.651(6)	6.843(5)	10.359(9)	91.55(2)
